# Nonlinear interference in crystal superlattices

**DOI:** 10.1038/s41377-020-0320-1

**Published:** 2020-05-09

**Authors:** Anna V. Paterova, Leonid A. Krivitsky

**Affiliations:** Institute of Materials Research and Engineering (IMRE), Agency for Science Technology and Research (A*STAR), 138634 Singapore, Singapore

**Keywords:** Quantum optics, Nonlinear optics, Quantum optics, Nonlinear optics

## Abstract

Nonlinear interferometers with correlated photons hold promise to advance optical characterization and metrology techniques by improving their performance and affordability. These interferometers offer subshot noise phase sensitivity and enable measurements in detection-challenging regions using inexpensive and efficient components. The sensitivity of nonlinear interferometers, defined by the ability to measure small shifts of interference fringes, can be significantly enhanced by using multiple nonlinear elements, or crystal superlattices. However, to date, experiments with more than two nonlinear elements have not been realized, thus hindering the potential of nonlinear interferometers. Here, we build a nonlinear interferometer with up to five nonlinear elements, referred to as superlattices, in a highly stable and versatile configuration. We study the modification of the interference pattern for different configurations of the superlattices and perform a proof-of-concept gas sensing experiment with enhanced sensitivity. Our approach offers a viable path towards broader adoption of nonlinear interferometers with correlated photons for imaging, interferometry, and spectroscopy.

## Improving interferometry with photon pairs from nonlinear crystals

Ultra-sensitive interferometric measurements can be made by using correlated photons generated in multiple nonlinear crystals, researchers in Singapore have shown. Correlated photons, linked through a form of quantum entanglement, are useful for studying materials in detection challenging spectral ranges. For example, when infrared photons are affected by striking a sample, the same changes will happen to their correlated photons of visible light, which are easier to detect. Anna Paterova and Leonid Krivitsky at A*STAR in Singapore developed an interferometer that generates correlated photons using up to five nonlinear elements which form crystal superlattices – periodic layers of different materials. Compared to previous systems that used only two nonlinear elements, the new system allowed the researchers to observe smaller changes in interference patterns, and therefore showed greater sensitivity when detecting carbon dioxide gas in tests.

## Introduction

Optical characterization and metrology techniques benefit from using correlated photons, particularly in studies of light-sensitive and fragile biological and chemical samples^1,2^. For example, strong temporal correlations between photons were used for a single-photon calibration of the efficiency of retinal cells^3^ and enhancing the nonlinear response of biological samples^4^. Furthermore, two-photon interference effects have formed the basis for dispersion-free optical coherence tomography^5–7^, microscopy with enhanced phase contrast^8,9^, and noise-robust spectroscopy of nanostructures^10^ to name a few.

Recently, the nonlinear interference of correlated photons has attracted particular interest in the context of infrared (IR) metrology and sensing^11–15^. A nonlinear interferometer is composed of two nonlinear elements, which produce pairs of correlated photons (signal and idler) under coherent excitation. The signal (in the visible range) and idler (in the IR range) photons are mixed in the interferometric setup, and as long as one cannot distinguish which nonlinear element produced the photons, interference fringes are observed. The interference pattern of signal photons depends on the phases and amplitudes of the signal, idler, and pump photons. When idler photons interact with a sample, its properties in the IR range can be inferred from the interference pattern of signal photons in the visible range. Thus, this technique addresses practical challenges of generation and detection of IR light since the sample response is obtained using accessible components for visible light.

Nonlinear interferometers have been realized using numerous physical platforms, including bulk nonlinear crystals^11–14,16–18^, gas cells^19^, fiberized networks^20,21^, and nonlinear waveguides^22,23^. Additionally, nonlinear interferometers have been used for imaging^24^, spectroscopy^16,25–27^, optical coherence tomography^28,29^, super-resolution interferometry^18,19^, and polarimetry^30^. All these techniques are intrinsically interferometric. Hence, their sensitivity is defined by the ability to detect small changes in the interference pattern, such as a shift of the fringes or change in the fringe visibility.

One possible way to enhance the sensitivity of nonlinear interferometers was outlined by D. Klyshko^31^, who considered a setup with *N* identical nonlinear elements separated by linear gaps, referred to here as a crystal superlattice. He showed that with an increase in the number of crystals, bright interference fringes in the frequency domain narrow, yet the spacing between fringes remains unchanged. This idea was theoretically expanded in more recent works^20,32,33^; however, to the best of our knowledge, there are no reports on the experimental realization of nonlinear interferometers with more than two nonlinear elements. The major challenges in practical realization are associated with (1) the necessity of superimposing signal and idler modes from multiple nonlinear elements while preserving the quantum indistinguishability, and (2) the necessity to align and stabilize increasingly complex setups.

Here, for the first time, we realize a nonlinear interferometer with a crystal superlattice consisting of up to five nonlinear elements. In our setup, nonlinear elements are arranged sequentially and are pumped by a single coherent laser. By careful design and alignment, we achieve a robust mode overlap of signal and idler photons with remarkable stability. We observe the interference pattern in the frequency-angular spectrum with full flexibility of crystal arrangements and theoretically describe this effect. We also perform a proof-of-concept gas sensing experiment with enhanced sensitivity.

## Results

### Theoretical framework

**Fig. 1:**
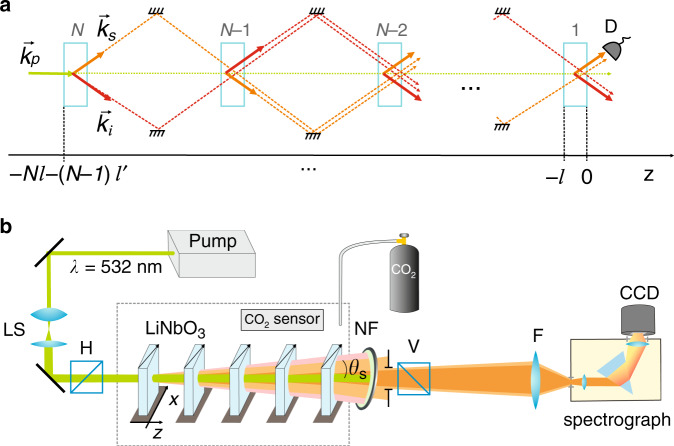
Nonlinear interferometer with crystal superlattice. **a** Conceptual scheme. *N* identical nonlinear crystals, separated by equal gaps, are coherently pumped by a laser (green arrow). In each crystal, the pump photon *k*_*p*_ decays into a pair of correlated signal *k*_s_ (orange arrow) and idler *k*_*i*_ (pink arrow) photons, which are then redirected to the next crystal. The intensity of signal photons is measured in the experiments by detector D. The paths of signal and idler photons are disjoined for clarity. **b** Experimental realization. A cw laser pumps SPDC crystals. Signal photons (orange) and idler photons (pink) generated in different crystals overlap within the interaction volume defined by the pump. The signal photons are projected by the lens F onto the slit of the imaging spectrograph with the CCD camera. The CCD camera captures the frequency-angular spectra. Optical axes of the crystals are aligned in the same direction (marked by the arrows). In the gas sensing experiments, carbon dioxide gas is injected into the enclosure (marked by a dashed rectangle).

Earlier works theoretically analysed the multicrystal interference in the frequency domain for a single spatial mode^31,34,35^. Analysis of the interference in both the frequency and spatial domains was limited to only two nonlinear crystals^36,37^. Here, we analyse the frequency-angular spectrum obtained in a nonlinear interferometer with *N* crystals. Let us consider *N* identical nonlinear crystals of length *l* separated by *N−1* equal linear gaps *l'*; see Fig. 1a. The crystals are pumped by a coherent laser, and each crystal produces signal (*s*) and idler (*i*) photons via spontaneous parametric down-conversion (SPDC). The down-converted photons from each crystal are redirected to the next crystal. The state of the two-photon field produced by a single crystal is given by^34^:

1 $$\left| \psi \right\rangle = \left| {{\mathrm{vac}}} \right\rangle + \mathop {\sum}\limits_n {\mathop {\sum}\limits_{k_s,k_i} {f_n\left( {\overrightarrow k _s,\overrightarrow k _i} \right)a_{k_{{\mathrm{ns}}}}^ + a_{k_{{\mathrm{ni}}}}^ + \left| {{\mathrm{vac}}} \right\rangle } }$$

where $f_n\left( {\overrightarrow k _s,\overrightarrow k _i} \right)$ is the two-photon field amplitude from the *n*-th crystal *n* = [1, *N*], $a_{k_{{\mathrm{ns}}}}^ +$ and $a_{k_{{\mathrm{ni}}}}^ +$ are creation operators of photons in the *n*-th crystal with wavevectors $\overrightarrow k _s$ and $\overrightarrow k _i$, respectively, and $\left| {{\mathrm{vac}}} \right\rangle$ indicates the vacuum state. Here, we assume that the photons are generated in the spontaneous regime and that the initial state of the signal and idler photons in each crystal can be considered a vacuum state^11,12,31^.

Assuming the pump is a monochromatic plane wave and the crystal is thin and uniform, the amplitude of a two-photon field is given by^31,38^:

2 $${f_n \propto \chi E_p\mathop {\int}\nolimits_{z_n}^{z_{n + l}} {dzD_n^{^\ast p}D_n^sD_n^i} }$$

where χ is the second-order susceptibility of the crystal; $z_n = - nl + \left( {n - 1} \right)l^\prime$ is the coordinate of the front edge of the *n*-th nonlinear crystal; *E*_*p*_ is the field of the pump; and $D_n^j$ is the following propagation function for the signal, idler, and pump photons (*j* = *s, i, p*):

3 $$D_n^j\left( {k_j,z} \right) = \exp \left[ { - ik_j^zz + \left( {n - 1} \right)\left( {k_j^{\prime z} - k_j^z} \right)l^\prime } \right]$$

where $k_j^z$ and $k_j^{\prime z}$ are the longitudinal wavevectors inside the nonlinear crystal and in the gap between crystals, respectively. From Eqs. (2) and (3), we obtain the two-photon field amplitude as follows:

4 $$f_n = \frac{{1 - \exp \left( { - i\Delta kl} \right)}}{{i\Delta kl}} \cdot \left[ { - i\left( {n - 1} \right)\left( {\Delta kl + \Delta k^\prime l^\prime } \right)} \right]$$

where Δ*k* and Δ*k'* are the wavevector mismatches inside the nonlinear crystal and in the linear gap, respectively. For *N* identical crystals, the two-photon field amplitude is given by the sum of contributions from individual crystals as follows:

5 $$F = \mathop {\sum}\limits_{n = 1}^N {f_n \propto {\mathrm{sinc}}\left( {\Delta kl/2} \right)\mathop {\sum}\limits_{n = 1}^N {e^{i\left( {n - 1} \right)\varphi }} } $$

where *φ* = (Δ*kl* + Δ*k'l*'). Then, from Eq. (5), the intensity distribution of the signal photons as a function of frequency *ω*_*s*_ and scattering angle *θ*_*s*_, as measured in the experiment, is given by^31,35,38^:

6 $${\mathrm{I}}_N\left( {\omega _s,\theta _s} \right) = \left| F \right|^2\, \propto \left\{ {{\mathrm{sinc}}\left( {\frac{{\Delta kl}}{2}} \right) \cdot \frac{{\sin \left[ {N\varphi /2} \right]}}{{\sin \left[ {\varphi /2} \right]}}} \right\}^2$$

**Fig. 2:**
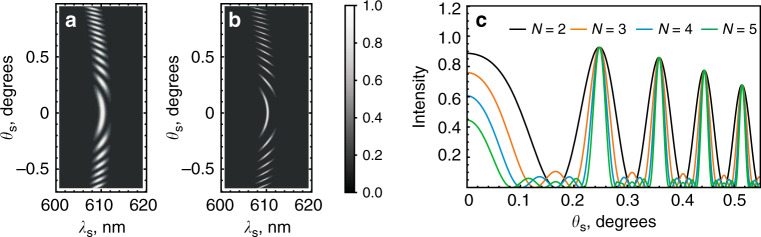
Theoretical results. Calculated frequency-angular spectra (interference patterns) of signal photons from two **a** and five **b** identical LiNbO_3_ crystals (crystal length *l* = 1 mm, air gaps between crystals *l’* = 8.2 mm, the orientation of the axis *θ*_c_ = 50.34°, the pump is a 532 nm laser). **c** Vertical cross sections of (**a**) and (**b**) at λ_s_ = 610.4 nm (idler photon wavelength is λ_*i*_ = 4142 nm) for different numbers of crystals in the superlattice.

We express the phase mismatch in the frequency and scattering angle in Section 1 of the Supplementary Materials^36,37^ and plot the interference patterns for the nonlinear interferometer with two and five crystals; see Fig. 2a, b, respectively. Figure 2c shows cross sections of the interference patterns for different numbers of crystals in the superlattice. We see that as the number of crystals increases, the interference maxima become narrower, yet the spacing between them remains unchanged.

In Section 2 of the Supplementary Materials, we show that the width of the bright fringes is inversely proportional to the number of crystals *N*:

7 $$\delta \theta _s \propto \frac{\pi }{N}$$

From Eq. (7), we note the striking similarity between the interference fringes for the nonlinear interferometer with a crystal superlattice and conventional multi-slit or Fabry-Perot linear interference^39^.

### Observation of the interference with a crystal superlattice

**Fig. 3:**
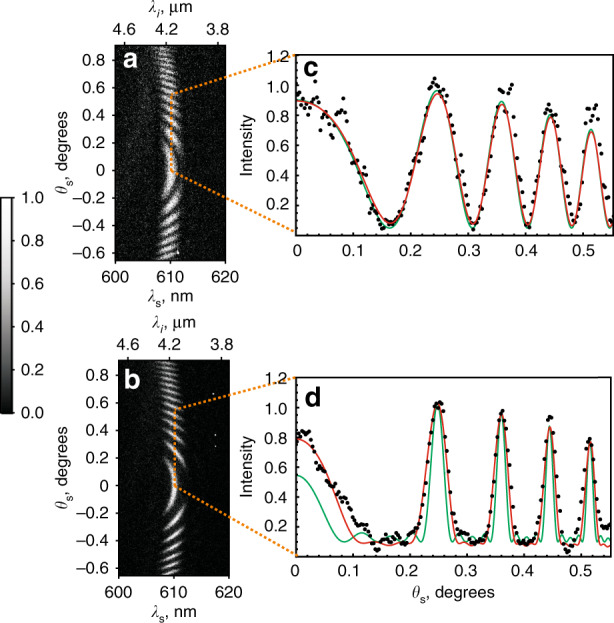
Experimental results. Normalized frequency-angular spectra (interference patterns) of signal photons for the nonlinear interferometer with **a** two and **b** five crystals. The bottom abscissa shows the detected wavelength of signal photons λ_s_, while the top abscissa indicates the wavelength of conjugated idler photons λ_*i*_. The greyscale for both graphs shows the intensity normalized to the maximum value in each experiment. **c**, **d** Cross sections of the interference fringes at λ_s_ = 610.4 nm for an interferometer with two (**c**) and five (**d**) crystals. Black dots are experimental data, and solid lines are theoretical calculations. The green curve in (**c**) and (**d**) shows calculations for the ideal case, and the red curve shows calculations taking into account the uncertainty in setting the phase-matching angle of each crystal by Δ*θ*_c_ = ±0.02°.

First, we set the phase-matching angle of the crystal to *θ*_c_ = 50.34° ± 0.02° when the signal SPDC photons are generated at ~610.4 nm (bandwidth 2 nm) and idler photons at 4.14 μm (bandwidth 92 nm). The normalized frequency-angular spectra of signal photons for two and five nonlinear crystals are shown in Fig. 3a, b, respectively. Our key observation is that the interference fringes for the interferometer with five crystals become narrower than those for the interferometer with two crystals, yet the period of the fringes remains unchanged. Figure 3c, d shows the cross sections of the interference pattern at λ_s_ = 610.4 nm for the interferometer with two and five crystals, respectively. The cross sections are taken by averaging the intensity across the bandwidth of Δλ_s_ = 0.4 nm. To achieve the same flux of the photons for the two- and five-crystal configurations, the acquisition time is set to 360 and 144 s, respectively.

The solid curves in Fig. 3c, d correspond to theoretical calculations. The green curve shows the theory in the ideal case, and the red curve shows the theory that accounts for the experimental accuracy in setting the phase-matching angle of each crystal to Δ*θ*_c_ = ±0.02°. We found that a slight misalignment in setting *θ*_c_ becomes crucial for the interferometer with an increasing number of crystals. A detailed analysis of the sensitivity of the interferometer to various experimental parameters is presented in Sections 3 and 4 of the Supplementary Materials.

**Fig. 4:**
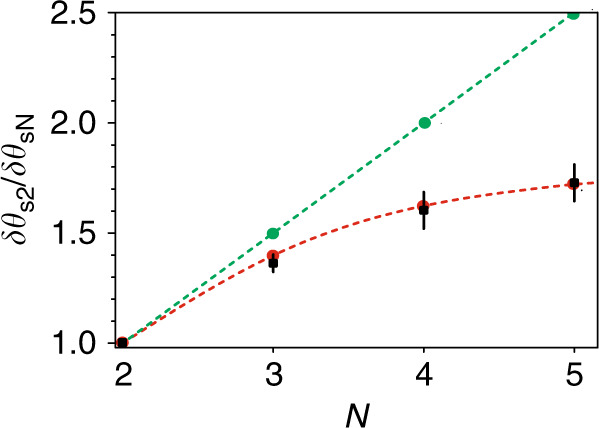
Scaling of the width of interference fringes. Experimental dependence of the ratio of the width of fringes δθ_s2_/δθ_sN_ on the number of crystals in the superlattice (black squares). The green dots show theoretical calculations in the ideal case; the red dots show the calculated dependence, which accounts for the experimental uncertainty in setting the phase-matching angle of *Δθ*_c_ = ±0.02°. Dashed lines are given to guide the eye.

We experimented with sets of two, three, four, and five crystals in the superlattice. In each case, we fit the experimental data by Eq. (6) and determined the width of the interference fringes. Figure 4 shows the ratio of the widths of the interference fringes for the *N-*crystal interferometer *δθ*_*sN*_ and two-crystal interferometer *δθ*_*s*2_. The key observation is consistent with the theory: the interference fringes become narrower with increasing number *N* of nonlinear crystals. The green dots in Fig. 4 show the linear scaling of the relative width in the ideal case; see Eq. (7). The red dots in Fig. 4 show the calculation results taking into account the uncertainty in setting the phase-matching angle of each crystal to Δ*θ*_c_ = ±0.02°, which is consistent with our experimental data, shown by black squares. Note that a stronger dependence on the uncertainty of the experimental parameters in the interferometer with a crystal superlattice is a manifestation of the common property of multielement interferometers.

### Proof-of-concept gas sensing experiment

**Fig. 5:**
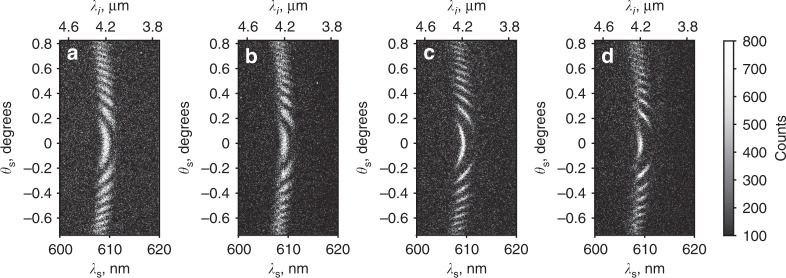
Results of the gas sensing experiment. The frequency-angular spectra (interference patterns) for the interferometer with two **a**, **b** and five **c**, **d** crystals. **a** and **c** show the reference interference pattern with the air gap between crystals, and **b** and **d** correspond to the data when CO_2_ gas is injected. Greyscale shows the CCD counts.

Next, we set the phase-matching angle to *θ*_c_ = 50.23° ± 0.02° and obtain signal photons at λ_s_ = 609.3 nm and idler photons in the vicinity of the absorption resonance of CO_2_ at λ_*i*_ = 4.19 μm. The frequency-angular spectra of signal photons from an interferometer with two and five crystals are shown in Fig. 5a–d, respectively. Figure 5a and c corresponds to the case when there is air in the gap between the crystals, and Fig. 5b and d corresponds to the case when CO_2_ gas is injected in the gaps (concentration (35 ± 3.5) × 10^3^ ppm). Because of the absorption of idler photons by the gas, the interference pattern of signal photons experiences a phase shift and reduction in visibility.

**Fig. 6:**
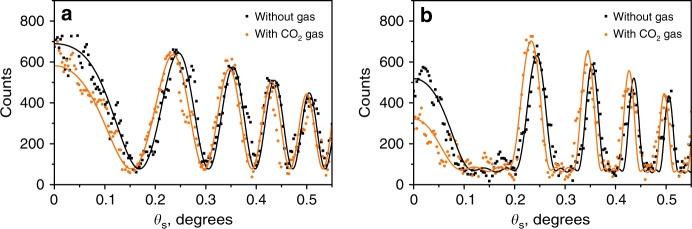
Analysis of interference fringes for the gas sensing experiment. Cross section of the interference patterns at λ_s_ = 609.3 nm for a nonlinear interferometer with **a** two and **b** five crystals. The black squares show the reference data with the air gap between crystals. Orange dots represent interference fringes with the injected CO_2_ gas. Black and orange lines show the fitting of the reference data and the data with injected CO_2_, respectively. The coefficient of determination (R^2^) for the two- and five-crystal interferometers is ~0.88 and 0.82, respectively. The vertical scale shows the CCD counts.

Figure 6 shows the cross sections of the interference pattern at λ_s_ = 609.3 nm (λ_*i*_ = 4.19 μm) when the wavelength of idler photons is detuned from the absorption resonance of CO_2_ by ~72 nm. In this case, the gas causes a phase shift of the interference fringes without a significant change in the fringe visibility. Figure 6a, b corresponds to the interference fringes for an interferometer with two and five crystals, respectively. The reference measurement is taken with air between the crystals. The points correspond to the experimental data; the solid lines show the fitting of the experimental data using Eq. (6).

From the fitting of the experimental data, we find that the relative shift of the interference fringes for the two-crystal interferometer is ${\Delta \varphi}_{2}$ = −(0.167 ± 0.015)π, and in the five-crystal interferometer, it is ${\Delta \varphi}_{5}$ = −(0.187 ± 0.009)π. Thus, the precision in the measurement of the phase shift in the five-crystal interferometer is 1.66 times higher than that in the two-crystal interferometer. This value is consistent with the reduction in the width of the bright interference fringe; see Fig. 4. The sensitivity of CO_2_ detection in the current five-crystal configuration is 1.8 × 10^3^ ppm.

The sensitivity of this method can be further improved by using more nonlinear crystals and increasing the interaction length. In practice, such a device can be realized using a high-finesse cavity with just a single crystal. We estimate that for a cavity with a finesse of 150 and a base of 80 mm, the theoretical value of the sensitivity reaches a level of a few tens of ppm, which is comparable with that of compact commercial optical sensors^40^.

### An interferometer with a “defect” in the superlattice

Our experimental setup allows full flexibility in investigating nonlinear interferometers with variable crystal configurations. To demonstrate this, we remove the third crystal from the interferometer and observe the interference from the first, second, fourth, and fifth crystals (assuming air between the crystals). We use Eq. (5) to calculate the interference pattern, which in this case is given by:

8 $$I \propto \left\{ {{\mathrm{sinc}}\left( {\frac{{\Delta kl}}{2}} \right) \cdot \cos \left[ {\Delta kl + \frac{{3\Delta k^\prime l^\prime }}{2} + \frac{{\Delta k^\prime l }}{2}} \right]\cos \left[ {\frac{{\Delta kl}}{2} + \frac{{\Delta k^\prime l^\prime }}{2}} \right]} \right\}^2$$

**Fig. 7:**
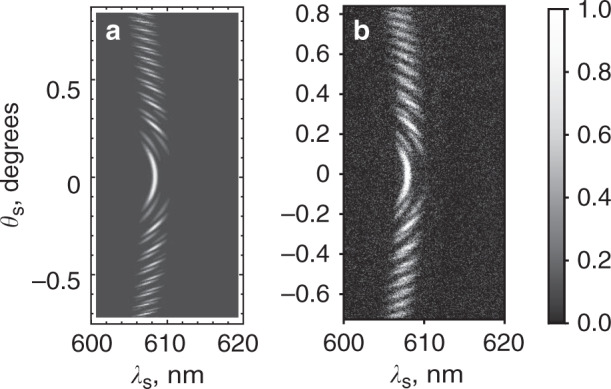
Interference in the superlattice with a “defect”. Normalized frequency-angular spectra (interference patterns) in the case when the third crystal is removed from the interferometer. The interference from the first, second, fourth, and fifth crystals is observed: **a** theoretical and **b** experimental dependencies. The parameters of the interferometer are similar to those in Figs. 6 and 7 with an air gap between the crystals. The data are normalized to the maximum counts of the CCD camera.

The theoretical interference pattern given by Eq. (8) is shown in Fig. 7a, and the corresponding experimental results are shown in Fig. 7b. The results are found to be in good agreement. As one can see, the interference pattern contains additional contributions originating from interferometers with different gaps. The ability to manipulate the interference patterns opens up possibilities for quantum state engineering^20,35^.

## Discussion

We realized a nonlinear interferometer with a crystal superlattice with up to five nonlinear elements. We experimentally demonstrated that with an increase in the number of nonlinear elements, the interference fringes become narrower, which directly translates to improved sensitivity in metrological and sensing applications. The observed effect originates from the constructive interference of wavefunctions of down-converted photons, which are coherently generated in different crystals. The effect is clearly analogous with classic multi-source interference. We also found that an interferometer with a crystal superlattice becomes increasingly dependent on the accuracy in setting the experimental parameters, in particular, the phase-matching angles of the crystals. This reflects the common property of multiple-beam interferometers, which are more demanding for the settings of individual elements.

The presented configuration allows flexibility in the realization of unconventional crystal configurations, for example, by setting different gaps between crystals and using crystals of different sizes, which opens an interesting possibility for quantum state engineering.

We anticipate that our work will trigger more than one creative design in the realization of complex nonlinear interferometers with correlated photons, such as by using mirrors, integrated photonics, or fibre platforms. It is also of interest to investigate this scheme for the high-gain regime of parametric down-conversion^41^. We believe that the presented concept will provide a viable path towards high-performance devices for sensing, metrology, and quantum state engineering. When finalizing this work, we became aware of relevant work on the realization of a three-stage nonlinear interferometer^42^.

## Materials and methods

Our experimental setup is shown in Fig. 1b. A continuous-wave laser (cw) with a wavelength of 532 nm (60 mW, Laser Quantum) pumps a set of identical lithium niobate nonlinear crystals cut from a single master crystal (5% MgO:LiNbO_3_, *l* = 1 mm, cut angle of 48.5°, Eksma Optics). Each crystal is coated with broadband AR coating, which introduces less than 1% loss for pump and signal photons and less than 5% loss for idler photons. The crystals are separated by the distance *l’* = 8.2 mm. They are mounted on a kinematic prism mount (KM100PM Thorlabs) and clamped by a small adjustable clamping arm (PM3, Thorlabs). The mount provides 0.45 deg adjustment per revolution. The estimated experimental accuracy in setting the phase-matching angle of the crystal is Δ*θ*_c_ = ±0.02°.

Photon pairs are generated in each nonlinear crystal in the type-I quasi-collinear frequency nondegenerate regime. A notch filter NF and a polarizer V are used to filter out the pump. Signal photons are focused on the slit of the imaging spectrograph (Acton) using the lens F (*f* = 300 mm). The interference pattern of signal photons in frequency-angular coordinates is recorded by a CCD camera for visible light (Andor iXon 897) at the output of the spectrometer. The camera has 512 × 512 pixels and a pixel size of 16 μm, the gain of the camera is set to 290, and the temperature of the camera sensor is kept at −80 °C. The optical noise is measured independently and taken into account at the stage of data processing.

To ensure the indistinguishability of photon pairs produced in every crystal of the superlattice, all the SPDC photons should be generated and propagate within the interaction volume defined by the pump beam. This requirement is expressed in the condition (2 *l* + *l’*ʹ)tan(*θ*_s_) *≪* *d*, which links the scattering angle *θ*_s_, pump diameter *d*, and parameters of the superlattice *l, l*ʹ(ref. ^16,26^). To satisfy this condition, we set *d ~* 3 mm using the beam expander (LS) and detect angles up to *θ*_s_ = ±0.85°.

Obtaining interference patterns with high visibility requires careful alignment of the interferometer. First, the orientation of each crystal is set to generate identical frequency spectra, which are measured by the spectrograph; see Section 5 of the Supplementary Materials. Then, by observing pairwise interference fringes between crystals, we ensure that the optical axes of the crystals are aligned in the same direction. Next, the distances between the crystals are carefully aligned to ensure equal gaps between them; see Sections 3 and 6 of the Supplementary Materials. Each crystal is mounted on a 2D translation stage so that it can be moved in and out of the interferometer. By successively observing the interference patterns from two, three, and four crystals, we adjust the distances between the crystals such that the fringes overlap. The accuracy of setting the length of the gap *l’* between crystals by this method is better than 100 μm. After the alignment of the crystals, we perform measurements of the interference with different numbers of crystals in the superlattice. The influence of the crystal length and the gap between the crystals on the interference pattern is analysed in detail in Section 7 of the Supplementary Materials.

In the gas sensing experiments, the interferometer is placed in an airtight enclosure (marked by a dashed rectangle) with an input socket for carbon dioxide gas (CO_2_, 99.9% purity). The wavelength of the idler photons is set to match the absorption peak at ~4.27 μm. The gas homogeneously fills the volume between the crystals. Its concentration in the enclosure is controlled by a commercial CO_2_ sensor (Amphenol, accuracy ±10% of reading). The experiments are conducted at a room temperature of 22 °C.

## Supplementary information

Supplementary Information for Nonlinear interference in crystal superlattices
