# Supplementary material for: Nonlinear interference in crystal superlattices
Source: Light Sci Appl. 2020 May 9;9:82. doi: 10.1038/s41377-020-0320-1 (PMC7211232; doi:10.1038/s41377-020-0320-1)
Supplement: Supplementary file 1 — Supplementary Information for Nonlinear interference in crystal superlattices [file 41377_2020_320_MOESM1_ESM.docx]

**Supplementary Information for:**

**Nonlinear interference in crystal superlattices**

By Anna V. Paterova, and Leonid A. Krivitsky*

*Institute of Materials Research and Engineering (IMRE),*

*Agency for Science Technology and Research (A*STAR), 138634 Singapore*

*e-mail: [Leonid_Krivitskiy@imre.a-star.edu.sg](mailto:Leonid_Krivitskiy@imre.a-star.edu.sg)

1. **Frequency-angular dependence of the interference pattern**

The frequency-angular spectrum of the SPDC is calculated using the phase-matching conditions:

$\omega_{p}=\omega_{s}+\omega_{i}$, (A1.1a)

$\Delta\vec{k}=\vec{k}_{p}-\vec{k}_{s}-\vec{k}_{i}$, (A1.1b)

where *ω_p,s,i_* are the frequencies of the pump, signal and idler photons.

The wavevector mismatch $\Delta\vec{k}$ has only a longitudinal component, while the transverse component is assumed to be zero (crystal is considered infinite in the transverse direction). Therefore, we can write the following equations:

$\Delta k=k_{p}-k_{s}\cos\theta_{s}^{\mathrm{int}}-k_{i}\cos\theta_{i}^{\mathrm{int}}$, (A1.2a)

$k_{s}\sin\theta_{s}^{\mathrm{int}}=k_{i}\sin\theta_{i}^{\mathrm{int}}$, (A1.2b)

where $\theta_{s,i}^{\mathrm{int}}$ are the internal scattering angles of the signal and idler photons. Using Eq. A1.2a, the wavevector mismatch $\Delta k$ inside the crystal can be defines as:

$\Delta k=\frac{2\pi}{c}\left(n_{p}\omega_{p}-n_{s}\omega_{s}\cos\theta_{s}^{\mathrm{int}}-n_{i}\omega_{i}\cos\theta_{i}^{\mathrm{int}}\right)$, (A1.3)

where $n_{p,s,i}$ are the refractive indices of the nonlinear crystal. A similar equation can be obtained for the wavevector mismatch $\Delta k'$ in the gap between crystals:

$\Delta k'=\frac{2\pi}{c}\left(n'_{p}\omega_{p}-n'_{s}\omega_{s}\cos\theta_{s}-n'_{i}\omega_{i}\cos\theta_{i}\right)$, (A1.4)

where $n'_{p,s,i}$ are the refractive indices the medium in the gap, $\theta_{s,i}$ external scattering angles of signal and idler photons. Form Eq. A1.1a and A1.2b we can write:

$\omega_{i}\cos\theta_{i}^{\mathrm{int}}=\left(\omega_{p}-\omega_{s}\right)\sqrt{1-\sin^{2}\theta_{i}^{\mathrm{int}}}=\left(\omega_{p}-\omega_{s}\right)\sqrt{1-\left(\frac{n_{s}\omega_{s}\sin\theta_{s}^{\mathrm{int}}}{n_{i}\left(\omega_{p}-\omega_{s}\right)}\right)^{2}}$. (A1.5)

We rewrite equations in terms of the external angel $\theta_{s}$, using Snell’s law of refraction. Then, summarizing Eqs. A1.3-A1.5, the final frequency-angular dependences of $\Delta k$ and $\Delta k'$ are given as follows:

$\Delta k\left(\omega_{s},\theta_{s}\right)=\frac{2\pi}{c}\left(n_{p}\omega_{p}-n_{s}\omega_{s}\sqrt{1-\left(\frac{\sin\theta_{s}}{n_{s}}\right)^{2}}-n_{i}\left(\omega_{p}-\omega_{s}\right)\sqrt{1-\left(\frac{\omega_{s}\sin\theta_{s}}{n_{i}\left(\omega_{p}-\omega_{s}\right)}\right)^{2}}\right)$, (A1.6a)

$\Delta k'\left(\omega_{s},\theta_{s}\right)=\frac{2\pi}{c}\left(n'_{p}\omega_{p}-n'_{s}\omega_{s}\sqrt{1-\left(\frac{\sin\theta_{s}}{n'_{s}}\right)^{2}}-n'_{i}\left(\omega_{p}-\omega_{s}\right)\sqrt{1-\left(\frac{\omega_{s}\sin\theta_{s}}{n'_{i}\left(\omega_{p}-\omega_{s}\right)}\right)^{2}}\right)$. (A1.6b)

These expressions are used for calculating the intensity distribution of the signal photons as a function of frequency *ω_s_*, and scattering angle *θ_s_* for signal photons, see Eq. 6 in the main text.

**2. Calculation of the width of the interference fringes**

The maxima of the interference pattern in Eq. 6 are observed, when ,$m=\left[0,\text{ }\pm1,\text{ }\pm2\text{ ... }\pm\infty\right)$. The positions of the intensity minima are determined by the fast oscillating function and observed at , $m'=\left[\pm1,\text{ }\pm2\text{ ... }\pm\infty\right)\cup m'\neq mN$. The width of the interference fringes is determined as the difference between positions, where interference reaches maxima and the closest minima ($m'=mN+1$):

. (A2.1)

As the phase mismatches $\Delta k$ and $\Delta k'$ are proportional to the square of the detection
angle $\theta_{s}$, Eq. A2.1 can be expressed as the following:

. (A2.2)

Expanding Eq. A2.2 and using Eq. A2.1, we obtain the following:

, (A2.3)

where $\delta\theta_{s}$ is the width of the interference fringes in angular coordinates. Hence, the width of the fringes is inversely proportional to the number of nonlinear crystals in the interferometer:

$\delta\theta_{s}\propto\frac{\pi}{N\left(\theta_{s,\max}+\theta_{s,\min}\right)/2}=\frac{\pi}{N\left\langle\theta_{s}\right\rangle}$, (A2.4)

where$\left\langle\theta_{s}\right\rangle$is an average of $\theta_{s,\max}$ and $\theta_{s,\min}$. Note that the width of the fringes decreases with the detection angle, which can be clearly seen from the measurements.

Form Eq. A2.4 the ratio of the width of the interference fringes in two- and *N*-crystal interferometer is given by:

$\frac{\delta\theta_{s2}}{\delta\theta_{sN}}\propto\frac{N\left\langle\theta_{sN}\right\rangle}{2\left\langle\theta_{s2}\right\rangle}\approx\frac{N}{2}$, (A2.5)

here where we assume that $\left\langle\theta_{s2}\right\rangle\approx\left\langle\theta_{sN}\right\rangle$.

**3. Sensitivity of the interference pattern to the uncertainty in the distance between the crystals.**

We experimentally tested the sensitivity of the interference pattern for an interferometer with five crystals to a slight misalignment of the distance between the fourth and the fifth crystals. The misalignment of the gaps by 100 μm affects the visibility of the interference fringes only at larger angles, see Fig. S1.

*
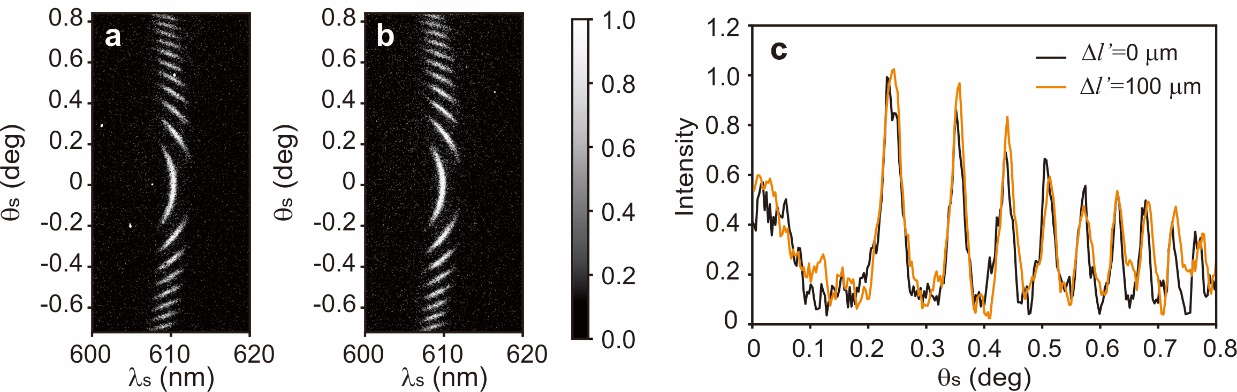
*

***Fig. S1****. Alignment of the gap between five crystals.* ***a*** *Interference pattern, when all gaps are aligned* Δ*l’=0 μm.* ***b*** *Interference pattern, when the fifth crystal is misaligned by* Δ*l’=100 μm.* ***c*** *Cross-sections of figures* ***a*** *and* ***b*** *at* Δλ_s_*=610 nm.*

**4. Sensitivity of the interference pattern to the uncertainty in the crystal length, the gap width, and the orientation**

We calculated the theoretical interference pattern for an interferometer with five crystals (*θ*_c_=50.34°, *l*=1 mm, *l’*=8.2 mm) by varying the value of the crystal length Δ*l*, the gap between crystals Δ*l’=100 μm,* and the phase-matching angle Δ*θ*_c_ (see Fig. S2). We found that the slight misalignment in the setting of the phase-matching angle within Δ*θ*_c_=±0.02°, which corresponds to our experimental accuracy, becomes a key factor, which is responsible for the broadening of the interference fringes.

*
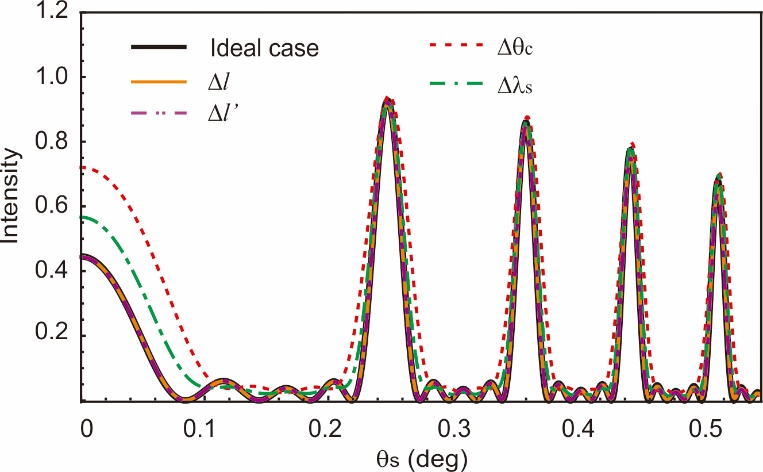
*

***Fig. S2****. Theoretical calculations of the interference fringes in the interferometer with five crystals for an ideal case (solid black curve) and a small variation in crystal length* Δ*l=±0.1 mm (solid orange curve), gap length* Δ*l’=100 μm (dash-dotted purple curve), phase matching angle* Δ*θ*_c_ *=0.02*° *for each crystal (dashed red curve). Dash-dotted green curve is calculated by averaging over the wavelength bandwidth of* Δλ_s_*=0.4 nm. The uncertainty in the setting of the phase-matching angle is a critical factor that affects the interference pattern.*

**5. Spectral alignment of crystals in the superlattice**

Crystals used in the experiment are cut from the same slab of the nonlinear material. The orientation of each crystal is set to generate identical frequency spectra, which are measured by the spectrograph. Each crystal is adjusted separately by moving in and out of the optical path. The measured spectra of individual crystals are shown in Fig. S3.

**
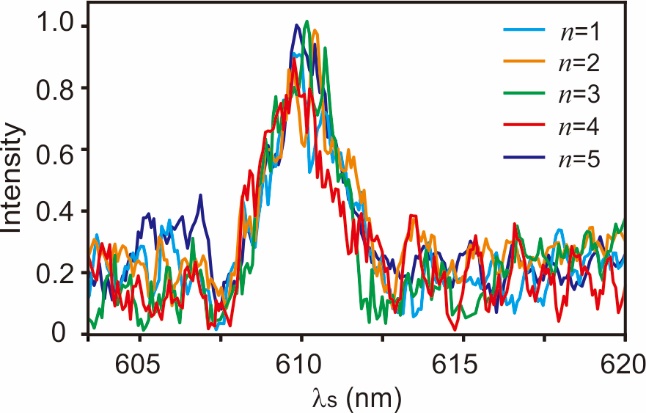
**

***Fig. S3****. The spectrum of signal photons from n-th crystal taken at θ_s_=0*°*. The orientation of each crystal is adjusted so that the spectra from all the crystals coincide.*

**6. Alignment of the orientation and the gap between the crystals**

Distances between the crystals are carefully aligned to ensure equal gaps between them. Each crystal can be moved in and out of the interferometer. By successively observing interference patterns from the interferometer with two, three, and four crystals, we adjust the distances between the crystals such that the fringes are overlapped, see Fig. S4.

*
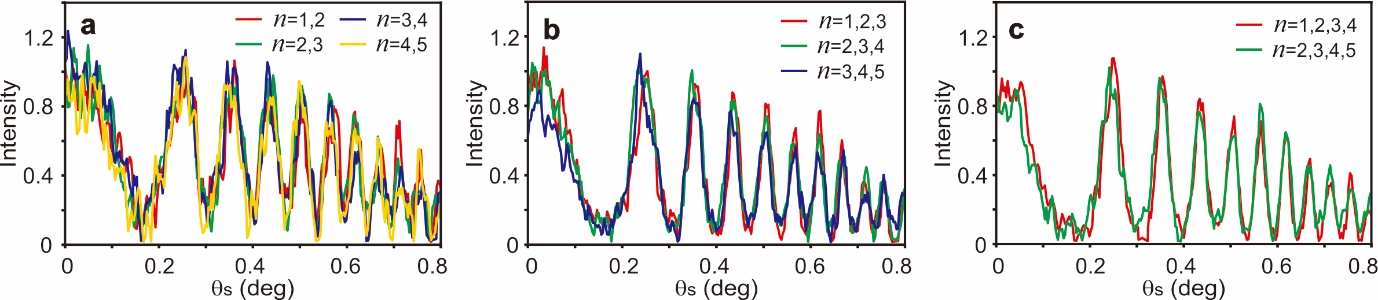
*

***Fig. S4****. Interference fringes from different combinations of* ***a*** *two,* ***b*** *three, and* ***c*** *four nonlinear crystals. The distance between the crystals is adjusted so that the interference fringes are in phase.*

**7. Effect of the gap and the crystal thickness on the interference pattern**

Fig. S5 shows the simulation results of the interference pattern for a nonlinear interferometer with five crystals for different values of the gaps *l’* and the crystal thicknesses *l*. The gap between the crystals defines the modulation period of the fringes: longer gap length leads to a high-frequency modulation in the angular coordinate (see Fig. S5a,b). The thickness of the crystals defines the spectral width of the SPDC (see Fig. S5b,c).


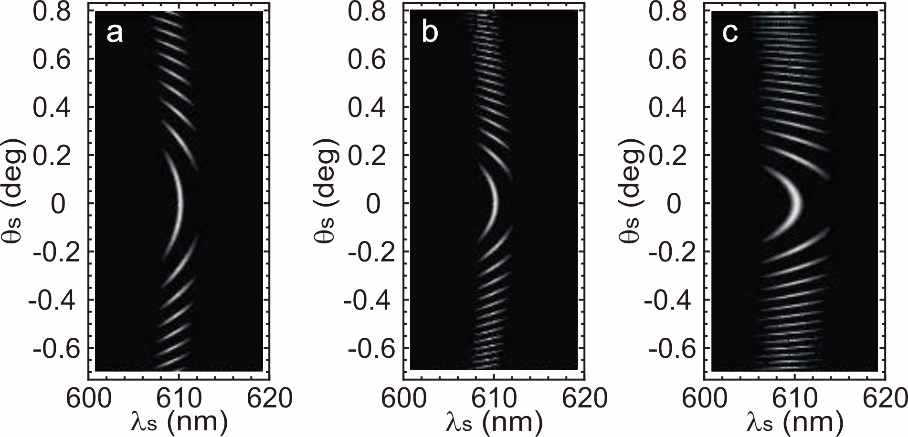


***Fig. S5****. Interference pattern from five-crystal interferometer for* ***a*** *l, l’,* ***b*** *l, 2l’, and* ***c*** *0.5l, 2l’. The parameters of the interferometer are the same as in the experiment: l=1 mm, l’=8.2 mm, and θ*_c_*=50.34°.*
